# Safranal Alleviates Cyclophosphamide Induced Testicular Toxicity in Rats

**DOI:** 10.1002/fsn3.70452

**Published:** 2025-06-16

**Authors:** Mustafa Cellat, İlker Yavaş, Ahmet Uyar, Muhammed Etyemez, Mehmet Güvenç

**Affiliations:** ^1^ Department of Physiology, Faculty of Veterinary Medicine Hatay Mustafa Kemal University Hatay Turkey; ^2^ Department of Reproduction and Artificial Insemination, Faculty of Veterinary Medicine Hatay Mustafa Kemal University Hatay Turkey; ^3^ Department of Pathology, Faculty of Veterinary Medicine Hatay Mustafa Kemal University Hatay Turkey; ^4^ Department of Physiology, Faculty of Veterinary Medicine Kastamonu University Kastamonu Turkey

**Keywords:** cyclophosphamide, male infertility, safranal, testicular toxicity

## Abstract

Safranal, the principal component of *Crocus sativus* essential oil, is primarily responsible for the characteristic aroma and distinct odor of saffron. Cyclophosphamide (CP), a chemotherapeutic agent commonly used in the treatment of both malignant and non‐malignant conditions, is known to induce cytotoxicity in various tissues, particularly within the male reproductive system. This study aimed to investigate the protective effects of safranal against CP‐induced reproductive toxicity in Wistar albino rats. CP was administered orally at a dose of 15 mg/kg once per week for 56 days to establish a model of testicular toxicity. In parallel, the treatment group received safranal via oral gavage at a daily dose of 200 mg/kg for the same duration. At the end of the treatment period, spermatological, biochemical, and histological analyses were performed on collected tissue samples. CP administration led to increased dead/live and abnormal sperm ratios, elevated levels of NF‐κB, IL‐6, TNF‐α, and MDA, and a reduction in sperm motility and density, Nrf‐2 expression, as well as GSH and GSH‐Px activity. In contrast, safranal treatment significantly ameliorated these detrimental effects. In conclusion, safranal demonstrated protective and therapeutic effects against CP‐induced reproductive toxicity, suggesting its potential as a supportive agent during chemotherapy.

## Graphical abstract

Cyclophosphamide, a chemotherapeutic agent, triggers inflammatory (TNF‐α, IL‐6, NF‐κB) and oxidative stress pathways, leading to impaired spermatogenesis and damage to germ cells in rats. Safranal appears to exert its protective action by activating the Nrf2 signaling pathway, thereby reducing oxidative stress and preserving normal spermatogenic architecture.

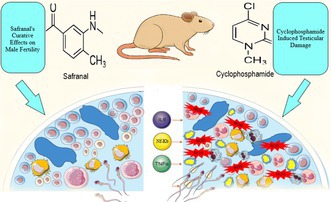

## 1 Introduction

Cyclophosphamide (CP) is an active alkylating agent in the nitrogen mustard family. It works by introducing alkyl radicals into cell DNA chains and inhibiting cancer cell proliferation, making it one of the most effective cancer treatments. It also has an immunosuppressive effect as it suppresses the body's natural immunological response (De Jonge et al. 2005; Perini et al. 2007). It is used to treat chronic and acute leukemias, multiple myeloma, lymphomas, and rheumatoid arthritis and also used for bone marrow transplantation preparation (Emadi et al. 2009; Nelius et al. 2010). CP has negative effects in numerous systems, including reproductive toxicity despite its vast variety of uses (Anan et al. 2018). The use of CP harms both healthy and malignant tissues, including the testes, bladder, and liver (Cengiz et al. 2020). CP is converted to phosphoramide mustard and acrolein by the microsomal cytochrome P450 enzyme in the liver. Acrolein is linked to CP's adverse effects and inhibits the antioxidant mechanism in cells, resulting in the formation of reactive oxygen species (MacAllister et al. 2013). The antineoplastic impact of CP is due to phosphorylated mustard, while the adverse effects are due to acrolein (Kern and Kehrer 2002). Acrolein suppresses the antioxidant system and causes cells to produce reactive oxygen species (MacAllister et al. 2013). Acrolein causes oxidative stress in normal cells, which causes DNA damage and toxicities in numerous target organs. It quickly penetrates the cell and triggers intracellular reactive oxygen species, which destroy the cell's lipids, proteins, and DNA. By promoting the production of free radicals in cells, it disrupts the GSH redox pathway (Kwolek‐Mirek et al. 2009). The exact mechanism of CP‐induced testicular toxicity has yet to be determined. CP exposure, on the other hand, has been found to disrupt redox balance, raising the possibility of biochemical and physiological abnormalities as a result of oxidative stress (Wang et al. 2007). It has been reported that CP causes testicular atrophy, and that oxidative stress, increased endoplasmic reticulum (ER) stress, and apoptosis are involved in the mentioned effect of molecular mechanism (Salama et al. 2020). Although CP is an effective chemotherapy for germ cell tumors, it causes transient or permanent azoospermia and oligospermia and disruption of hormonal status (Potnuri et al. 2018). Complex communication between different cell types in the testicles such as Sertoli, Leydig, and spermatogenic cells ensures normal spermatogenesis. Interactions between cells are regulated by paracrine and autocrine factors such as hormones, growth factors, proteins, and enzymes. Anticancer medications have been demonstrated to cause testicular dysfunction by altering the release of these factors, causing DNA damage, and hindering the germ cell repair process (De Flora et al. 1996; Stukenborg et al. 2018). Nrf2 (NF‐E2‐related factor 2) is a transcription factor that aids in the removal of unwanted intracellular reactive oxygen species and enhances antioxidant capacity (Li et al. 2012). Cells establish a series of signal regulating systems to maintain intracellular redox balance in order to minimize damage caused by CP or other oxidative stressors (Miao et al. 2005). One of these crucial pathways is Nrf2/ARE. Nrf‐2 is kept at extremely low levels in the cytoplasm under normal circumstances by binding to Keap1 or by rapid degradation. Drugs or other oxidative stressors can enhance the dissociation of Nrf‐2, releasing it from Keap1 and upregulating the expression of the Nrf‐2 gene. Once released, Nrf‐2 enters the nucleus, binds to the ARE pathway of genes, and subsequently activates the expression of genes involved in phase II enzymes, antioxidant enzymes, and protein kinases. Nrf‐2, which is widely distributed across various organs, plays a critical role in organ protection. Its deletion or active degradation makes cells more vulnerable to oxidative stress (Stępkowski and Kruszewski 2011). CP application has been shown to lower Nrf‐2 levels in mouse testicular tissue and increase oxidative stress (Le et al. 2015). In rats, it has been reported that CP administration led to significant increases in dead and abnormal sperm counts, as well as elevated protein carbonyl and lipid peroxidation levels. Additionally, CP caused significant decreases in sperm count, motility, antioxidant enzyme activities such as superoxide dismutase (SOD), catalase (CAT), and glutathione peroxidase (GSH‐Px), as well as a reduction in GSH levels (Selvakumar et al. 2006).

Several studies have shown that antioxidant agents can modulate chemotherapy toxicity while minimizing adverse effects (MacAllister et al. 2013). Cultivated in Southwest Asia, Spain, France, Italy, Turkey, and Iran, *Crocus sativus* L. is a flowering plant used in traditional medicine. At least 150 compounds are found in saffron, which comes from the flower of *Coriandrum sativum* L. the major components of saffron, crosin and safranal, have been shown to have anti‐inflammatory, anticonvulsant, antitussive, antioxidant, anxiolytic, and antidepressant properties (Razavi and Hosseinzadeh 2015). Safranal is one of saffron's key active ingredients and is responsible for the spice's antioxidant properties. Safranal stabilizes cell membranes, inhibits peroxidation of unsaturated membrane lipids, and scavenges ROS. Accordingly, safranal could be useful to defeat radical scavenging activity is important, such as in neuro illnesses (Samarghandian et al. 2015). Emerging evidence suggests that testicular toxicity and systemic oxidative stress may be modulated via gut–testis axis interactions. Functional foods like safranal could alter gut microbiota composition, impacting testicular immune responses (Ciernikova et al. 2025).

No study has been found in the literature indicating the effects of safranal against CP‐induced testicular toxicity. Based on this, the aim of this study was to investigate the potential protective effects of safranal in the CP‐induced testicular toxicity model.

## 2 Materials and Methods

### 2.1 Experimental Animals and Ethics Statement

A total of 32 Wistar albino male rats (8–10 weeks old, 250–300 g) were obtained from Hatay Mustafa Kemal University Medical Laboratory Animal Centre, Turkey. The rats were housed in a controlled environment with a temperature of 20°C–22°C, proper ventilation, a 12‐h light/dark cycle, and relative humidity of 50% ± 10%. All mice were given standard commercial pellet food (Carfil) and water ad libitum. All animals were handled in accordance with the European Union Directive 2010/63/EU on the protection of animals used for scientific purposes and in compliance with the ARRIVE (Animal Research: Reporting of In Vivo Experiments) guidelines and approved by the Local Animal Experiments Ethics Committee at Hatay Mustafa Kemal University (Approval Number: 2021/02‐17).

### 2.2 Experimental Design

The animals were randomly divided into four groups (*n* = 8 per group) as follows: *Group 1* (control group), *Group 2* (CP), *Group 3* (safranal), and *Group 4* (CP + safranal) were the four groups in our study. The cyclophosphamide dose for the experimental testicular toxicity model in rats was based on the study by Çeribaşi et al. (2010), while the safranal dose was selected according to the study by Karafakıoğlu et al. (2017).

First group (*control group*): Rats given isotonic water 1 mL with oral gauge for 56 days. Second group (*CP*): Rats were given 15 mg/kg of cyclophosphamide once a week for 56 days, dissolved in 1 mL of water. Third group (*safranal*): Rats in this group were administered safranal (200 mg/kg), dissolved in 1 mL of water, for 56 days by oral gauge. Fourth group (*cyclophosphamide* + *safranal*): Rats in this group were given safranal at 200 mg/kg in 1 mL of water for 56 days by oral gauge. Additionally, they received cyclophosphamide (15 mg/kg) in 1 mL of water per week for 56 days. No animals or data were excluded from the experiment/analysis. Blood samples were collected from the tail veins of all groups 24 h after the final administration. Euthanasia was then performed using the decapitation method. The testicular tissues of decapitated rats were extracted, and tissue samples were cleaned with lactated Ringer's solution. Blood serums were prepared after centrifuging the blood samples.

### 2.3 Spermatozoa Analysis

The procedure described by Turk et al. (2007) was used to conduct the spermatological examinations. Hemocytometry was used to determine the concentration of spermatozoa in the right caudal epididymal tissue. Sperm motility was assessed using freshly isolated left caudal epididymal tissue. A phase‐contrast microscope (Nikon E 200) equipped with a heating plate set at 37°C was employed to evaluate sperm motility percentage. To assess sperm morphology, samples were prepared using Hancock solution. Number of 400 sperm per preparation (2800 cells in total) were analyzed, and the overall abnormality rate was calculated as a percentage.

### 2.4 Biochemical Analysis

The levels of malondialdehyde (MDA) and glutathione (GSH), along with the activity of catalase (CAT) and glutathione peroxidase (GPx) enzymes, were assessed spectrophotometrically in tissue samples. Testicular tissue was homogenized with a 1/10 ratio of 1.15% KCl, and MDA analysis was conducted on half of the homogenate. The remaining homogenate was centrifuged at 5000 *g* for 1 h (+4°C), and the supernatant was collected for GSH measurement and enzyme activity analysis of GPx and CAT. Protein levels in both the homogenate and supernatant were determined using the Lowry method, and the results were normalized to the protein content (Aebi 1983; Lawrence and Burk 1976; Lowry et al. 1951; Placer et al. 1966; Sedlak and Lindsay 1968).

### 2.5 Testosterone Levels

In accordance with the protocols, commercial ELISA kits were used to determine serum testosterone levels (BT Lab Bioassay Technology Laboratory, Zhejiang Province, China).

### 2.6 Inflammatory Cytokines Analysis

Protein levels of TNF‐α, IL‐1β, IL‐6, and COX‐2 were assessed by ELISA method in accordance with the commercial kit procedure to determine the inflammation status in testicular tissue (BT Lab Bioassay Technology Laboratory, Zhejiang Province, China).

### 2.7 Western Blotting Analysis

The tissues were homogenized in cold RIPA lysis buffer (sc‐24948A), centrifuged at 14,000 rpm at +4°C, and the supernatant was collected following the standard protocol. Total protein levels in the tissues were then quantified performing the BCA method (Smith et al. 1985) with the commercial Kit. Equal amounts of protein (40 μg) were loaded into each well and electrophoresed on a polyacrylamide gel (Laemmli 1970). Following SDS‐PAGE (Towbin et al. 1979) proteins were transferred to a polyvinylidene difluoride (PVDF) membrane using the western blotting technique. The membranes were then incubated overnight with primary antibodies diluted in 5% milk powder: Nrf‐2 (CST 12721; rabbit monoclonal antibody; 1:1000), NF‐κB (CST 8242; rabbit monoclonal antibody; 1:10,000), and β‐actin (CST 4970; rabbit monoclonal antibody; 1:5000). Following incubation, the membranes were washed three times for 5 min each with TBS‐T. The membranes were treated with secondary antibodies (CST 7074 1/2000) for 1 h after the secondary antibodies (CST 7074 1/2000) were diluted with 5% milk powder. After incubation, the membranes were washed three times with TBS‐T for 5 min each. The bands, detected using chemiluminescent conjugate (ECL; Biorad, 1705061), were visualized on the chemiluminescence imaging system (Biorad ChemiDoc MP) (Bass et al. 2017; Sambrook et al. 1989). Band intensities in the images were quantified using the appropriate analysis system (Biorad Image Lab Software version 5.2.1; Biorad Laboratories Inc., USA). The data were normalized to β‐actin, which served as an internal control, and expressed as a percentage of the control.

### 2.8 Histopathological Analysis

At the end of the treatment, the testicular tissues of the rats were collected. The tissue samples were first placed in Bouin's fixative for 24 h, followed by fixation in a 10% buffered formaldehyde solution for 48 h and cleared in a xylene series before being embedded in paraffin. Hematoxylin–eosin‐stained sections of testicular tissue samples from each rat were examined with the guidance of a light microscope. A total of 200 seminiferous tubules per testis were analyzed across multiple fields, and mean Johnsen scores were calculated. The results were then evaluated and scored based on the Johnsen Testicular Biopsy Score method, as described in Table 1 (Johnsen 1970). Microphotographs were taken to document the findings. According to this method, the normal testis has a mean score of 8–10, with lower values indicating damage to the germinal epithelium and impaired germinative cell development and spermatogenesis.

**TABLE 1:** Characterization of the Johnsen testicular score.

10	The germinal epithelium is stratified around the central lumen, the lumen is open and contains many spermatozoa
9	Although germinal epithelium is seen, insignificant amount of debris that can wash the lumen, spermatozoa and rash epithelium in the occluded lumen
8	Multilayered germinal epithelium but less than 10 spermatozoa in the lumen
7	Numerous spermatids and no spermatozoa
6	Absence of spermatozoa and less than 10 spermatids
5	Presence of only spermatocytes, no spermatozoa or spermatids
4	Absence of any spermatozoa and spermatids and less than 5 spermatocytes
3	Presence of only spermatogonia as germ cells
2	Only Sertoli cells and no germ cells
1	No cells in the seminiferous tubule lumen

### 2.9 Statistical Analysis

The Shapiro–Wilk normality test was performed to determine whether the collected data followed a normal distribution, and the results confirmed normality for all parameters. Sample size was calculated using G*Power based on expected effect size of 0.8, *α* = 0.05, and power = 0.80, resulting in *n* = 8 per group. Group means were compared using one‐way analysis of variance (ANOVA), and Tukey's test was applied to identify differences between groups. Statistical analysis was conducted using the IBM SPSS Statistics 23 software, with *p* < 0.05 considered statistically significant.

## 3 Results

### 3.1 Spermatological Findings

Table 2 shows the spermatological results. The sperm motility and density, which had significantly decreased after cyclophosphamide treatment, significantly increased in the safranal group, reaching the same levels as the control group (*p* < 0.05). The abnormal sperm ratio and the percentage of dead/live spermatozoa (*p* < 0.05), both of which significantly increased after CP application, have no statistical difference with the safranal‐treated and control groups.

**TABLE 2:** Mean ± SEM values of sperm parameters.

Group/parameter	Concentration (million/right cauda epididymis)	Motility (%)	Anormal sperm (%)	Dead/live rate
Control	142,200 ± 4.76^a^	79,000 ± 1.87^c^	13,400 ± 0.74^a^	16,600 ± 1.36^a^
Cyclophosphamide	105,000 ± 3.46^b^	15,000 ± 1.58^a^	26,200 ± 1.56^c^	51,400 ± 2.11^c^
Safranal	128,600 ± 1.86^a^	79,000 ± 4.00^c^	17,400 ± 1.36^ab^	18,400 ± 1.02^a^
Cyclophosphamide + safranal	107,800 ± 2.98^b^	51,000 ± 4.30^b^	19,600 ± 1.07^b^	24,600 ± 1.28^b^
*p*	0.000	0.000	0.000	0.000

*Note:* The difference between values with different letters (a–c) in the same column is statistically significant.

### 3.2 Biochemical Findings

Table 3 presents the oxidative stress markers and antioxidant activity levels measured at the end of the treatment. The results indicate that MDA levels in the safranal‐treated group showed no statistically significant difference compared to the control group. However, in the CP group, cyclophosphamide administration led to a significant increase in MDA levels (*p* < 0.05). Similarly, GSH levels (*p* < 0.05) and GSH‐Px activities (*p* < 0.05) significantly decreased following cyclophosphamide administration. In contrast, no significant differences were observed between the safranal‐treated and control groups in terms of GSH levels (*p* < 0.05) or GSH‐Px activities (*p* < 0.05), suggesting that safranal may help maintain these antioxidant markers.

**TABLE 3:** Mean ± SEM values of malondialdehyde (MDA), reduced glutathione (GSH) levels and glutathione peroxidase (GSH‐Px), and catalase (CAT) in testis.

Group/parameter	MDA (nmol/mL)	GSH (nmol/mL)	GSH‐Px (IU/g prot)	CAT (U/mL)
Control	9061 ± 0.61^ab^	2155 ± 0.12^b^	27,690 ± 1.88^bc^	31,700 ± 0.93
Cyclophosphamide	13,349 ± 0.41^c^	1640 ± 0.,08^a^	17,198 ± 0.83^a^	28,979 ± 4.43
Safranal	7400 ± 0.53^a^	2231 ± 0.15^b^	30,092 ± 1.25^c^	29,483 ± 1.78
Cyclophosphamide + safranal	10,680 ± 0.56^b^	2138 ± 0.04^b^	23,078 ± 0.74^b^	29,461 ± 4.52
*p*	0.000	0.006	0.000	0.938

*Note:* The difference between values with different letters (a–c) in the same column is statistically significant.

### 3.3 Serum Testosterone Levels

**FIGURE 1:**
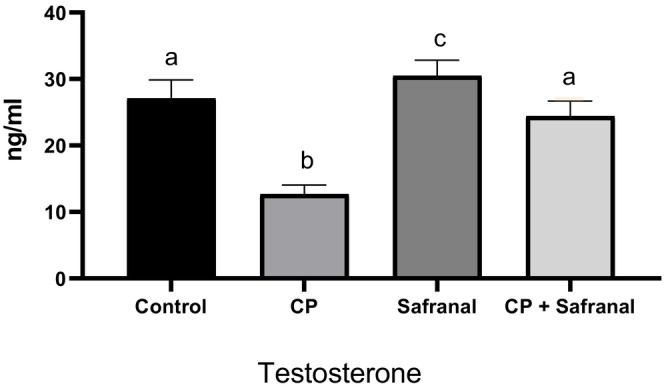
Serum testosterone levels of control and experimental groups. Data are expressed as mean ± SEM. Different letters (a–c) indicate statistical differences (*p* < 0.001).

Figure 1 presents data on serum testosterone levels acquired at the end of the trial. As a result, serum testosterone levels, which were significantly decreased by cyclophosphamide administration, in the safranal‐treated group have no statistical difference from the control group.

### 3.4 Inflammatory Cytokines Levels

Table 4 shows the findings related to the ELISA parameters obtained at the end of the treatment. Accordingly, TNF‐α (*p* < 0.05) and IL‐6 (*p* < 0.05) levels, which boosted with CP application, significantly decreased with safranal application, and have no statistical difference from the control group.

**TABLE 4:** Mean ± SEM values of TNF‐α, IL‐1β, IL‐6 and COX‐2 levels in testis.

Group/parameter	TNF‐α (nmol/mL)	IL‐1β (nmol/mL)	IL‐6 (IU/g prot)	COX‐2 (U/mL)
Control	75,540 ± 1.37^a^	359,433 ± 9.19	3549 ± 0.06^a^	1988 ± 0.05
Cyclophosphamide	90,940 ± 2.78^b^	377,866 ± 7.65	4012 ± 0.06^b^	1856 ± 0.05
Safranal	74,960 ± 2.23^a^	355,000 ± 5.12	3467 ± 0.08^a^	1911 ± 0.06
Cyclophosphamide + safranal	79,250 ± 0.55^a^	368,733 ± 8.56	3634 ± 0.09^a^	1768 ± 0.05
*p*	0,000	0,206	0.001	0.061

*Note:* The difference between values with different letters (a–c) in the same column is statistically significant.

### 3.5 Western Blot Parameters

**FIGURE 2:**
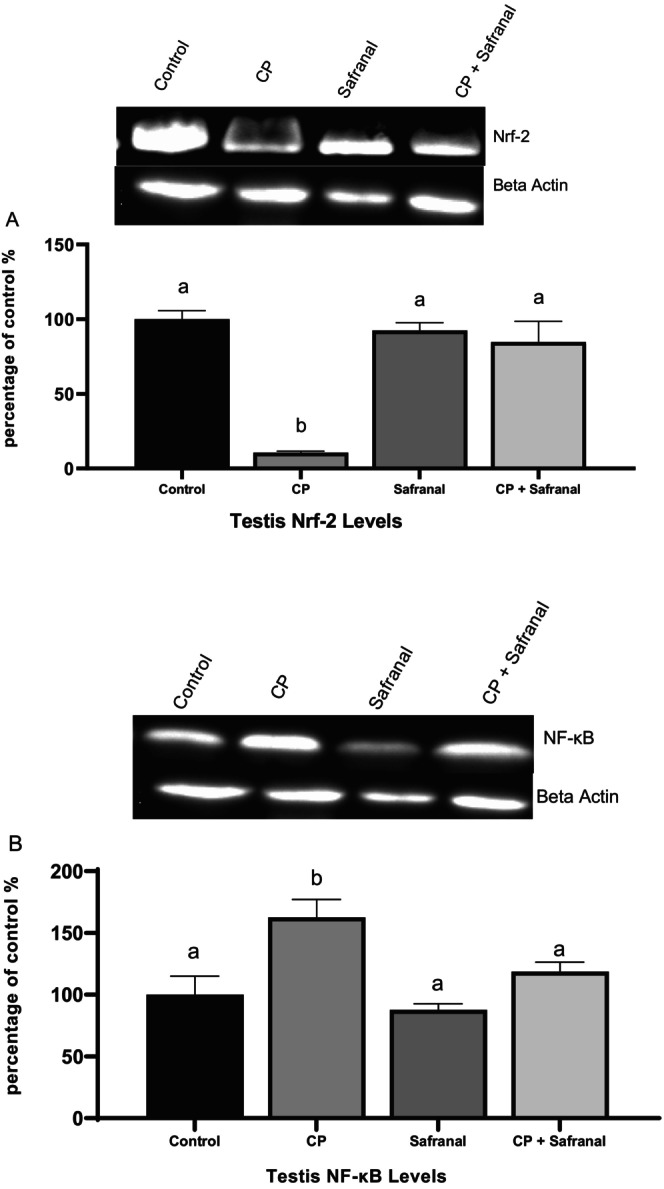
Effect of safranal on western blot analysis of Nrf‐2 and NF‐κB in the testis tissue of control and experimental rats. Protein expression is normalized to β‐actin. Data are shown as percent of control. (A) Representative western blot picture of Nrf‐2 and β‐actin in testis. Mean Nrf‐2 expression levels (%). (B) Representative western blot picture of NF‐κB and β‐actin in testis. Mean NF‐κB expression levels (%). Data are expressed as mean ± SEM. Different letters (a, b) indicate statistical differences. (*p* < 0.05).

Figure 2 shows the western blot results acquired at the end of the treatment. Nrf‐2 expression in the safranal group showed no statistical difference compared to the control group (*p* < 0.05), despite a significant decrease following cyclophosphamide administration. In the safranal‐treated group, however, Nf‐kB expression, which increased with CP application, have no statistical difference with the control group.

### 3.6 Histopathological Findings

**FIGURE 3:**
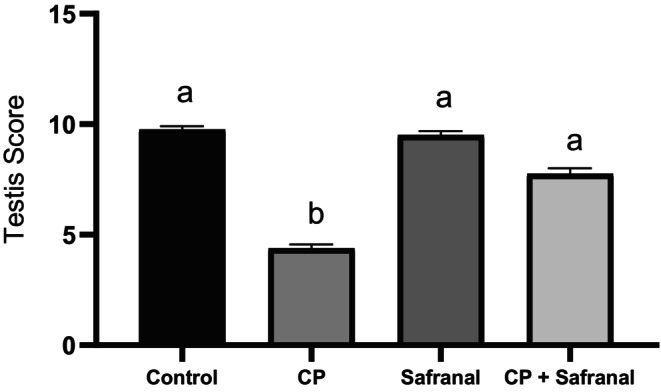
Histopathologic variables in testes according to the John's score in study groups. Data are expressed as mean ± SEM. Different letters (a, b) indicate statistical differences (*p* < 0.001).

**FIGURE 4:**
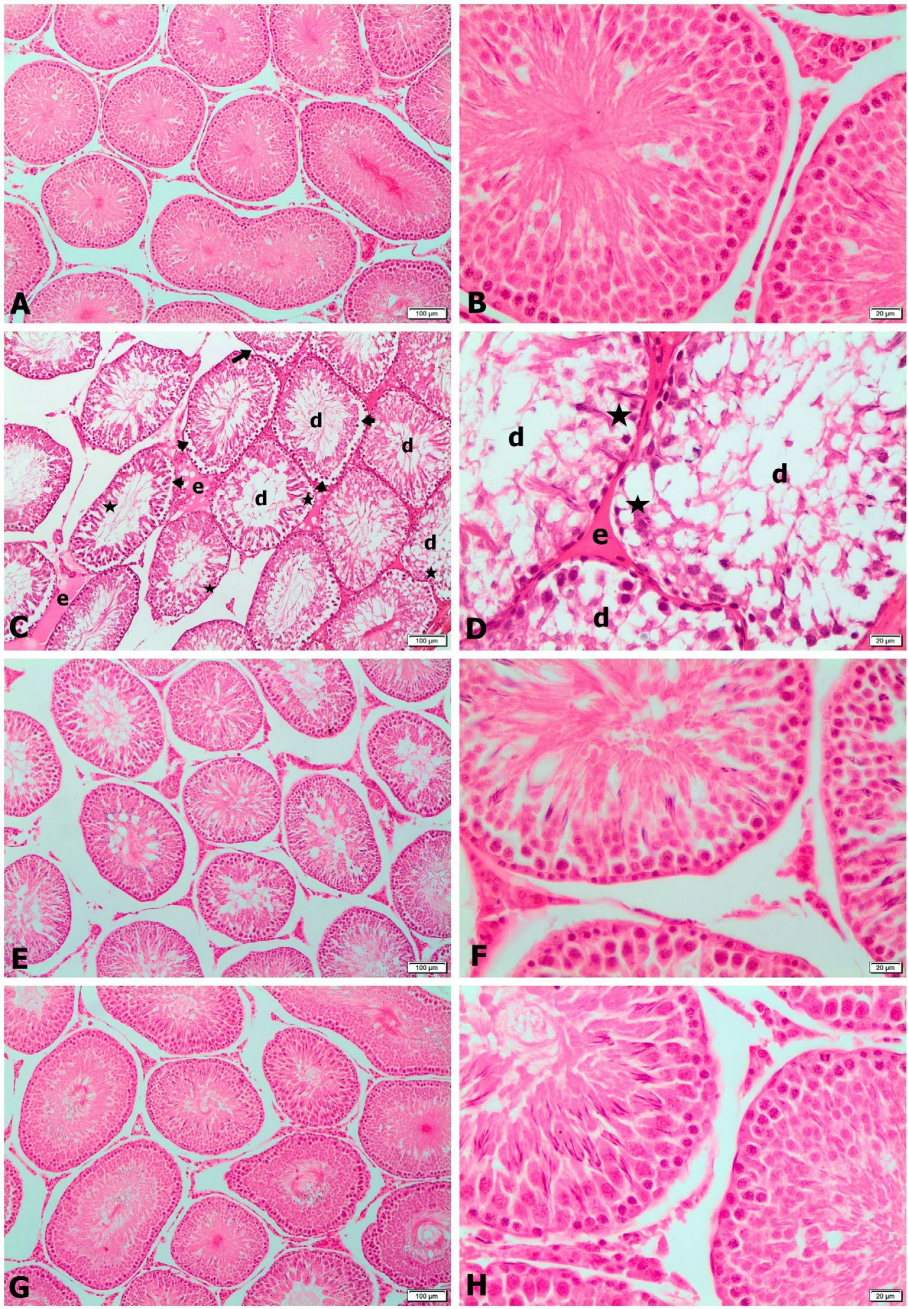
Testicular sections were stained with hematoxylin and eosin dye and examined using light microscopy at magnification ×100 (A, C, E, G) and ×400 (B, D, F, H). (A, B) Control group; normal histological appearance of testicular tissue. (C, D) Cyclophosphamide group; severe degeneration of seminiferous tubules (d), separation of peribasal germinative epithelial cells from the basement membrane (arrow heads), degenerative changes in germ cells and decrease in cell number (asterisk), undulation (arrow) and microscopic appearance of edema (e). (E, F) Cyclophosphamide + safranal group; near normal histological appearance of testicular tissue. (G, H) Safranal group; normal histological appearance of testicular tissue.

Figure 3 shows the mean scores of testicular tissue samples stained with hematoxylin–eosin and graded by Johnsen's scoring under a light microscope, whereas Figure 4A–H shows microscopic images of histological results.

When the mean Johnsen scores were compared between the groups, the Cyclophosphamide group had the lowest mean value. When the groups were evaluated in terms of mean score, the control and safranal groups had similar scores, which have no statistical difference (*p* > 0.05). When the control and CP groups were examined, it was found that the CP + safranal group has no statistical difference with the control group (*p* > 0.05), whereas the cyclophosphamide + safranal group was statistically different (*p* < 0.05) from the CP group.

Light microscopy analysis showed that the seminiferous tubules' morphological structure and basement membrane, as well as the interstitial area containing Sertoli and spermatogenic cells, and Leydig cells, all displayed a normal histological structure in the testes of both the control group (Figure 4A,B) and the safranal group (Figure 4G,H). Spermatogenesis was observed to continue in the normal course in these groups' seminiferous tubules.

Light microscopic examination of testicular tissue from rats treated with CP revealed severe damage to the seminiferous tubules and interstitial area (Figure 4C,D). Some tubules exhibited disrupted and degenerated morphology, while others appeared adjacent in certain areas. Almost all series cells, from spermatagonium cells forming the parenchyma structure of seminiferous tubules to spermatozoa, showed severe degenerative changes and a significant decrease in cell numbers. Many seminiferous tubules were found to be made up entirely of spermatogonia cells, which are sparse roof cells, and Sertoli cells, with very few spermatogonia seated on the basement membrane. The stages of spermatogenesis ceased in the majority of seminiferous tubules. The germinative cell layer thickness was negligible in these tubules. The lumens of such tubules were found to be empty, and the spermatozoa, which were few in the lumens, were also degenerated. Germinative cells in the peribasal area of the seminiferous tubules were also separated from the basement membrane, and some basement membranes showed corrugation. Edema was observed in the interstitial area, and Leydig cells appeared reduced in number and deviated from their normal histological structure (Figure 4C,D).

In the testes of CP + safranal group rats, the majority of seminiferous tubules preserved their morphology and had an almost normal histological appearance, with only a few showing slight damage. Moreover, tubular damage was significantly reduced, germinative cells were preserved, and normal spermatogenesis appeared to continue. In the interstitial area, Leydig cells exhibited a normal histological structure, with moderate edema observed in certain regions (Figure 4E,F).

## 4 Discussion

This study investigated the protective effects of safranal against cyclophosphamide (CP)‐induced reproductive toxicity in an experimental model. To this end, inflammatory cytokines, oxidative stress parameters, Nrf‐2 and NF‐κB protein expressions, spermatological variables, and histopathological changes were evaluated. To the best of our knowledge, this is the first study to report the protective potential of safranal against CP‐induced male reproductive toxicity.

Among chemotherapeutic agents, alkylating agents are known to exert the most pronounced gonadotoxic effects in males (Meistrich 2009; Oktay and Meirow 2007). CP, a cytotoxic bifunctional alkylating agent from the nitrogen mustard family, is particularly noted for its severe gonadal toxicity (van der Kaaij et al. 2010). It primarily targets testicular cells involved in the early stages of steroidogenesis and spermatogenesis. Consequently, testosterone levels and spermatological parameters serve as essential indicators of testicular damage (Tates 1992). In both humans and rats, CP has been shown to cause oligospermia or aspermia due to structural and biochemical alterations in the testis and epididymis (Meistrich 2009). This is largely attributed to acrolein, a toxic CP metabolite, which promotes lipid peroxidation and excessive ROS production by impairing the antioxidant defense system (Mythili et al. 2004). Such oxidative damage induced by acrolein leads to apoptosis and cell death in testicular tissue, ultimately contributing to male infertility (Kern and Kehrer 2002).

Several studies have shown that CP administration negatively affects sperm quality, including motility, density, and morphology (Kim et al. 2013; Oyagbemi et al. 2016; Shabanian et al. 2017). These impairments are closely linked to disruptions in testosterone‐mediated interactions between Sertoli and germ cells, leading to failed spermatogenesis (Higuchi et al. 2001). Furthermore, ROS‐mediated inhibition of steroidogenic enzymes such as 17‐β hydroxysteroid dehydrogenase and 3‐β hydroxysteroid dehydrogenase results in reduced circulating testosterone and LH levels (Chandrashekar 2009). This hormonal imbalance contributes to impaired spermatogenesis and increased sperm abnormalities. In this study, CP exposure led to decreased serum testosterone levels, reduced sperm motility and density, and increased ratios of abnormal and dead/live sperm. These alterations are indicative of impaired spermatogenesis, primarily driven by oxidative stress. Reduced testosterone production and androgen receptor abnormalities caused by CP therapy may affect spermatogenesis and testicular functioning, leading to an increase in morphological sperm defects. In the CP group, we found a decrease in serum testosterone levels, a decrease in sperm motility and density, and an increase in the ratio of abnormal and dead/live sperm. The loss of sperm cells at various stages of development has been linked to the impairment in spermatological parameters, and the origin of these effects has been linked to oxidative damage (Higuchi et al. 2001). Polyunsaturated fats in testicular tissue and sperm membrane are highly reactive to reactive oxygen species (ROS), resulting in oxidative damage to germ cells, spermatozoa, and mature sperm (Vernet et al. 2004). Oxidative damage is known as the main cause of male infertility and testicular injury. Related mechanisms include oxidative stress in the testicles and changes in microvascular blood flow, which increases germ cell apoptosis (Turner and Lysiak 2008). The interaction of acrolein with protective antioxidant mechanisms is at the root of these consequences. Acrolein causes male infertility by inducing apoptosis in testicular tissues, in addition to its negative effects on male reproductive function (Kern and Kehrer 2002). Nutraceuticals with antioxidant and anti‐inflammatory activity—such as resveratrol, curcumin, or quercetin—show promise in counteracting testis damage and may offer chemopreventive effects. These agents act on various pathways including anti‐inflammatory and antioxidant effects (Marini et al. 2022, 2023; Požgajová et al. 2022). Safranal, similarly, may fit into this therapeutic landscape. The presence of elevated MDA and decreased antioxidants GSH and GSH‐Px, both of which are indicators of lipid peroxidation, confirmed cyclophosphamide‐induced testicular oxidative stress in this investigation. Various studies have also observed similar findings (Can et al. 2020; Ebokaiwe et al. 2021; Iqubal et al. 2020). However, in the safranal‐treated group, the oxidative damage was reduced, and antioxidant activity enhanced. Safranal is known to have protective effects against ROS buildup and the ability to prevent the peroxidation of unsaturated membrane lipids as a result of numerous investigations (Assimopoulou et al. 2005; Papandreou et al. 2011). The curative results shown in the current study may be due to these effects of safranal.

Nrf‐2 is a key transcription factor regulating antioxidant gene expression and is activated in response to oxidative stress to mitigate ROS accumulation and cellular damage. Activation of Nrf‐2 is also associated with suppression of the NF‐κB pathway, thus modulating inflammation (Li et al. 2008). In their work, Nakamura et al. (2010) stated that inhibition of Nrf‐2 leads to testicular oxidative stress and impaired spermatogenesis, highlighting its critical role in reproductive function. Similarly, Chen et al. (2020) reported a correlation between Nrf‐2 expression and spermatological parameters. Several studies have shown CP‐induced downregulation of Nrf‐2 in testicular tissue (Fusco et al. 2021; Maremanda et al. 2014). Consistent with these reports, our study found reduced Nrf‐2 expression in CP‐treated rats, which was restored by safranal treatment. The antioxidant effect of safranal may therefore be mediated, at least in part, by activation of the Nrf‐2 pathway (Arkali et al. 2021; Xia et al. 2019). The protective effect of safranal may involve modulation of MAPK and caspase signaling pathways, which are central to cellular responses to oxidative stress and apoptosis. Prior studies support this mechanism, indicating that therapeutic modulation of these pathways could underlie the observed molecular improvements (Almuqati 2025; Altavilla et al. 2012). The antioxidant properties of safranal are thought to activate the Nrf‐2 pathway in the current investigation.

Oxidative stress and inflammation are closely interlinked. ROS leakage during inflammation promotes leukocyte migration and tissue injury (Mittal et al. 2014). NF‐κB, a key transcription factor, regulates inflammatory responses by controlling the expression of cytokines such as TNF‐α, IL‐6, and COX‐2 (Farombi et al. 2009). Dysregulation of the NF‐κB pathway has been linked to various pathological conditions, such as cancer and inflammatory diseases (Moon et al. 2009). CP‐induced ROS may activate the NF‐κB signaling pathway, exacerbating inflammatory damage (Rezaei et al. 2021). On the other hand, inhibition of NFκB signaling in the group treated with safranal may be due to the free radical scavenging properties of safranal. Overactivation of the NF‐κB signaling pathway, a transcription factor that stimulates the release of proinflammatory cytokines, leads to the excessive release of inflammatory cytokines, including TNF‐α, IL‐1β, and IL‐6 (Al‐Massri et al. 2018; Ma and Kinneer 2002). TNF‐α, a crucial cytokine, is involved in the development of inflammatory toxicity produced by a variety of chemicals (Ma and Kinneer 2002). In this study, CP administration increased NF‐κB activity and upregulated TNF‐α and IL‐6 levels in testicular tissue, in line with previous studies (Nafees et al. 2015; Wang et al. 2021). TNF‐α and IL‐6 levels in rats given CP increased in our investigation, corroborating these findings. Reduced levels of these cytokines in the safranal‐treated group, on the other hand, revealed safranal's anti‐inflammatory potential, which is consistent with earlier research. However, safranal treatment significantly reduced these inflammatory markers, likely due to its free radical scavenging and anti‐inflammatory effects (Hazman and Ovalı 2015; Lertnimitphun et al. 2021). CP‐induced systemic inflammation is likely mediated by NLRP3 inflammasome activation. Safranal's anti‐inflammatory effects may include suppression of this pathway, suggesting a novel mechanism of chemoprotection (Sano et al. 2022).

The above‐mentioned conclusions are also supported by histopathological studies. A decrease in Johnsen testicular scores, atrophy and degeneration of the seminiferous tubules, atrophic Leydig cells, and interstitial edema were discovered during a histological examination of testicular toxicity. Pathological changes related to CP toxicity include a reduction in antioxidant enzyme activity and an upregulation of proteins (Senthilkumar et al. 2006). There were improvements in histological structure in the safranal‐treated group. The antioxidant and anti‐inflammatory properties of safranal are likely to be responsible for these therapeutic effects.

In conclusion, this study presents new insights into the protective mechanisms of safranal against CP‐induced testicular toxicity. CP induces oxidative stress and inflammation via activation of the NF‐κB pathway and suppression of Nrf‐2, resulting in impaired spermatogenesis, hormonal imbalance, and histological damage. Safranal counteracts these effects by enhancing Nrf‐2 activity and inhibiting NF‐κB signaling, thereby reducing ROS production and inflammatory cytokine expression. These findings support safranal as a promising candidate for mitigating chemotherapeutic‐induced reproductive toxicity. However, this study is limited by the lack of different pathways, immune staining, and absence of long‐term follow‐up. While results in rodent models are promising, clinical translation requires further validation. Human testicular physiology differs in sensitivity and recovery from toxicants, and dosage equivalence must be carefully considered. Moreover, while rat models are informative, extrapolation to humans requires caution due to interspecies differences. Further studies exploring different doses and treatment durations of safranal are warranted to optimize its therapeutic potential.

## Author Contributions

**Mustafa Cellat:** investigation (equal), methodology (equal), project administration (equal). **İlker Yavaş:** formal analysis (equal), investigation (equal), methodology (equal). **Ahmet Uyar:** formal analysis (equal), methodology (equal). **Muhammed Etyemez:** data curation (equal), formal analysis (equal), investigation (equal), methodology (equal). **Mehmet Güvenç:** conceptualization (equal), data curation (lead), funding acquisition (supporting), methodology (equal), project administration (equal), writing – original draft (lead), writing – review and editing (lead).

## Ethics Statement

Animal experiments approved by the Local Animal Experiments Ethics Committee at Hatay Mustafa Kemal University (Approval Number: 2021/02‐17).

## Conflicts of Interest

The authors declare no conflicts of interest.

## Data Availability

Authors elect not to share the data.
